# A Case Series Highlighting Complexities in the Management of Bipolar Disorder With Parkinson’s Disease

**DOI:** 10.1192/bjo.2025.10719

**Published:** 2025-06-20

**Authors:** Lawrence Dianga, Mathew Madu, Alana Durrant, Leia Penfold, Joanna Pepper

**Affiliations:** Norfolk and Suffolk Foundation Trust, Bury St Edmunds, United Kingdom

## Abstract

**Aims:** Evidence is emerging regarding the association between Bipolar Affective Disorder (BPAD) and Parkinson’s disease (PD). Studies have shown patients with BPAD have a higher risk of developing PD. This case series explores the complexities encountered in the management of patients with PD and BPAD.

**Methods:** ‘A’ is a woman in her 70s with BPAD. Parkinsonian symptoms were noted for several years, suspected to be medication-induced. She was diagnosed with Parkinson’s disease, after a positive DaT scan, treated with co-beneldopa, which induced a manic episode, requiring hospitalization. Co-beneldopa was stopped, she improved with lamotrigine and clonazepam.

‘B’ is a woman in her 70s with BPAD with a family history of Parkinson’s disease. She was hospitalized after relapse of BPAD. She was noted to have a unilateral tremor, stooped posture and a shuffling gait. She is now being assessed for Parkinson’s disease.

‘C’ is a woman in her 60s with BPAD, on sodium valproate and aripiprazole. She was reviewed by neurology due to bilateral tremor, rigidity and unsteady gait, and subsequently diagnosed with drug-induced parkinsonism. Due to miscommunication, GP started her on co-beneldopa, for Parkinson’s disease. Subsequently, she developed mania warranting hospitalization. The ward team was unaware of the misdiagnosis. Co-beneldopa was subsequently stopped. She continued having poor oral intake, intractable mania, treated with ECT.

**Results:** Literature review shows BPAD is associated with Parkinson’s disease (PD). Evidence indicates, a diagnosis of BPAD, increases the risk of developing PD. With no established intervention for patients with co-morbid BPAD and PD, treatment becomes complex. Proposed pathology suggests BPAD is exacerbated by heightened dopamine levels, while PD from reduced dopamine. This makes it challenging to treat one without impacting the other. Mood stabilizers and antipsychotics can contribute to drug-induced parkinsonism (DIP), which may clinically be indistinguishable from PD. Antiparkinsonian medications like dopamine agonist and pramipexole can cause manic symptoms.

**Conclusion:** Historically, heterogeneity in psychiatric disorders, both in presentation and response, remains the norm. In this case series, we try to highlight the complex relationship between BPAD and PD. To establish a direct causal relationship is challenging due to the various confounders. This being a niche topic with limited research, emphasizes the need for large sample studies, which could shed more light on the longitudinal course and relationship between the two disorders and help establish future treatment guidelines.

